# Mechanochemical Phosphorylation and Solubilisation of β-D-Glucan from Yeast *Saccharomyces cerevisiae* and Its Biological Activities

**DOI:** 10.1371/journal.pone.0103494

**Published:** 2014-07-30

**Authors:** Feng Shi, Jikui Shi, Yongfu Li

**Affiliations:** 1 State Key Laboratory of Food Science and Technology, Jiangnan University, Wuxi, China; 2 Key Laboratory of Carbohydrate Chemistry and Biotechnology, Ministry of Education, School of Biotechnology, Jiangnan University, Wuxi, China; 3 Synergetic Innovation Center of Food Safety and Nutrition, Jiangnan University, Wuxi, China; 4 National Engineering Laboratory for Cereal Fermentation Technology, Jiangnan University, Wuxi, China; University of Tokyo, Japan

## Abstract

To obtain a water-soluble β-D-glucan derivative cleanly and conveniently, a highly efficient mechanochemical method, planetary ball milling, was used to phosphorylate β-D-glucan isolated from yeast *Saccharomyces cerevisiae* in solid state. Soluble β-D-glucan phosphate (GP) with a high degree of substitution (0.77–2.09) and an apparent PEAK molecular weight of 6.6–10.0 kDa was produced when β-D-glucan was co-milled with sodium hexametaphosphate at 139.5–186.0 rad/s for 12–20 min. The energy transferred was 3.03–11.98 KJ/g. The phosphorylation of GPs was demonstrated by Fourier transform infrared spectroscopy and ^13^C and ^31^P Nuclear magnetic resonance spectroscopy. Three GP products with different degree of substitution (DS) and degree of polymerisation (DP) were able to upregulate the functional events mediated by activated murine macrophage RAW264.7 cells, among which GP-2 with a DS of 1.24 and DP of 30.5 exerted the highest immunostimulating activity. Our results indicate that mechanochemical processing is an efficient method for preparing water-soluble and biologically active GP with high DS.

## Introduction

β-D-glucan, a major component of yeast cell walls, is known as a potent immunostimulant and has significant augmenting effects on host defence systems [1]. The target immune cells of β-D-glucan include macrophages, neutrophils, monocytes, natural killer cells, and dendritic cells, with macrophages being the principal target cells [2]. The binding of β-D-glucan to these cells triggers phagocytosis and stimulates the release of a cascade of cytokines from macrophages, such as tumor necrosis factor (TNF-α) and various types of interleukins (ILs) [3]. Macrophages activated by cytokines secrete nitric oxide (NO), which is toxic to tumor cells and microorganisms [4]. Therefore, by enhancing the host immune function, β-D-glucan exhibits antimicrobial and antitumoral activities. A major obstacle to the clinical utilisation of β-D-glucan as a biological response modifier is its relative lack of solubility in aqueous media [5]. The application of insoluble β-D-glucans in clinical settings would cause significant adverse effects, such as hepatosplenomegaly, granuloma formation, microembolisation, inflammation, pain, and increased endotoxin sensitivity, when administered by parenteral routes [6], [7]. Therefore, the preparation of water-soluble β-D-glucans would be advantageous, as reported previously by Qin et al. [8].

To improve the water solubility of β-D-glucans, several methods have been developed. One type of method is the chemical modification of β-D-glucans via, for example, carboxymethylation [8]–[10], sulphation [11], and phosphation [5], [12]. Through chemical modification, hydrophilic groups are introduced into β-D-glucans and the water solubility of these β-D-glucans derivatives is improved. Although the polyelectrolyte formed through these modifications may have different biological properties compared to neutral β-D-glucans, some derivatives of β-D-glucans have been reported to exert high immunomodulatory activity. For example, the water soluble β-D-glucan phosphate can specifically bind to monocyte/macrophage cell lines, accelerate wound healing, and attenuate cardiac dysfunction [13], [14]. The water soluble β-D-glucan sulfate can activate macrophages, stimulate bone marrow, and exerts antitumor therapeutic activity [11], [15]. However, the degree of substitution (DS) and molecular weight influence the antitumor activities of the sulfated β-D-glucan [16]. The water soluble carboxymethylated β-D-glucan can exhibit mitogenic and antioxidative activity [17], [18]. Another type of method is the physical degradation of β-D-glucans, such as through ultrasonic depolymerisation [6]. However, the solubility of the weakly degraded β-D-glucans produced in this manner is not improved as greatly as they are by chemical modification. However, in almost all procedures used to chemically modify β-D-glucans, the reactions are commonly carried out in an organic solvent at elevated temperature under strongly acidic or alkali conditions; thus, the cost associated with solvent recycling and neutralisation and energy consumption are unavoidable. Therefore, it is necessary to develop a simple, clean, and efficient procedure for preparing water-soluble β-D-glucans derivatives.

“Mechanochemistry” offers opportunities for developing new and cleaner synthesis procedures with either no or only nominal amounts of added solvent [19]. Mechanochemistry refers to reactions, normally of solids, induced by the input of mechanical energy, such as by grinding in ball mills. During mechanochemical processing, some mechanical energy can be converted to internal energy in the milled solids, and thus, many metastable active sites can be generated, which facilitate the reaction of the solids with other reagents. Mechanochemistry has been applied in comprehensive fields, such as alloy and materials engineering and pharmacology [20], [21]. Recently, mechanochemical treatment was applied to induce morphological and structural development and to improve the physicochemical properties of some polysaccharides, including cellulose [22], [23], starch [24], and lignocelluloses [25]. A few reports have demonstrated that milled cellulose can react directly with co-milled reagents, such as acetic anhydride [26] and lauric acid [27], to produce cellulose derivatives, such as surface-acetylated cellulose powder and cellulose laurate, indicating mechanochemical processing as a prospective protocol for the solid-state modification of polysaccharides; however, the ball-milling process is typically time-consuming.

In the present study, to obtain a water-soluble β-D-glucan derivative cleanly and conveniently, highly efficient mechanochemical processing via planetary ball milling was applied to phosphorylate β-D-glucan isolated from brewer’s yeast, *Saccharomyces cerevisiae*, within 20 min. The obtained β-D-glucan phosphate (GP) was able to upregulate the functional events mediated by activated macrophages, including phagocytosis, production of cytokines, such as TNF-α and IL-6, and secretion of NO.

## Materials and Methods

### Preparation of β-D-glucan phosphate

The mechanochemical preparation of β-D-glucan phosphate was performed in a planetary ball mill (XXM, Jiaxing, China) equipped with two jars (150 mL each). The insoluble β-D-glucan isolated from brewer’s yeast, *S. cerevisiae*, was provided by Shanghai Gecono Yeast Co. Ltd. (Shanghai, China). The insoluble β-D-glucan particles contained 4% water, 1.5% protein, and 94% β-D-glucan among which about 80% was (1→3)-linked β-D-glucan and 20% was (1→6)-linked β-D-glucan. Before milling, β-D-glucan was mixed thoroughly with sodium hexametaphosphate at different weight ratios (1∶2, 1∶4, and 1∶6). Subsequently, 15 g of the mixture was added to one of the jars and milled with 150 g of balls (diameter = 3 mm) at a certain rotational speed (139.5 or 186.0 rad/s) for a certain time (12, 16, and 20 min). During the milling, the jars were cooled by a water bath. Then, the milled powder was dissolved in deionised water and centrifuged at 4000 rpm for 20 min to remove insoluble β-D-glucan. The supernatant was collected, dialysed against deionised water thoroughly to remove excess sodium hexametaphosphate until the elemental phosphorus could not be detected in the permeate of dialysis, and finally lyophilised to obtain the GP products at a maximal DS of 2.07. GPs with different DS could be easily produced by changing the rotational speed, milling time, and charge ratio.

### Evaluation of water solubility

The milled powder of β-D-glucan-hexametaphosphate (2.0000 g) was dissolved in 25 mL of deionised water and centrifuged at 4000 rpm for 20 min. The carbohydrate content of the supernatant and that of the initial milled powder were determined by the anthrone-sulphuric acid method [28]. The water solubility of β-D-glucan phosphate was calculated by the carbohydrate content of the supernatant divided by the carbohydrate content of the original milled powder.

### Determination of molecular weight by HPLC

The apparent molecular weight of β-D-glucan phosphate and native β-D-glucan (NG) was determined by high-performance liquid chromatography (HPLC, Waters 1525, Waters, USA). Eight milligrams of lyophilised GP and NG powder was dissolved in 2.0 mL of distilled water and dimethylsulphoxide (DMSO), respectively, and passed through a 0.23-*µ*m filter for HPLC analysis. HPLC was performed on two linked gel-filtration chromatographic columns of ultrahydrogel™ 500 (Waters, USA) (7.8×300 mm) eluted with 0.1 M NaNO_3_ at a flow rate of 0.9 mL/min and detected by a refractive index detector. The PEAK molecular weight of GP was calculated by the calibration curve obtained by using various standard dextrans (Dextran T20, T40, T100, and T200).

### Determination of the degree of substitution (DS) and degree of polymerisation (DP)

The DS of β-D-glucan phosphate was calculated by the content of elemental phosphorus according to the following formula [29], which yields the average number of phosphate groups on each glucose residue.
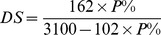



The amount of elemental phosphorus was detected by spectrophotometric method based on the formation of phosphomolybdate with added ammonium molybdate [30], using KH_2_PO_4_ as a standard. In this formula, P% is the percentage of phosphorus content of the β-D-glucan phosphate, 162 is the molar mass of the anhydroglucose unit, 3100 is the atomic weight of phosphorus multiplied by 100, and 102 is the molar mass of the phosphate substituent (−NaHPO_3_) subtracted by the molar mass of the substituted hydrogen atom (−H).

The degree of polymerisation (DP) of GP was calculated according to the formula:
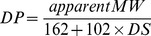



### Fourier transform infrared (FTIR) spectroscopy

FTIR spectroscopic analysis of the native β-D-glucan and β-D-glucan phosphate was performed using a Nicolet Nexus 470 FT-IR spectrophotometer (Nicolet, USA) with a resolution of 4 cm^−1^ and a frequency range of 4000–400 cm^−1^; 32 scans were performed for each sample [31]. Each sample was mixed with KBr thoroughly in a 1∶100 ratio and then pressed into a small pellet measuring approximately 1 mm thick.

### ^13^C and ^31^P Nuclear magnetic resonance (NMR) spectroscopy

^13^C and ^31^P NMR analysis of the native β-D-glucan NG and β-D-glucan phosphate GP was performed on an AVANCE-400 spectrometer (Bruker, Switzerland). Before analysis, NG and GP samples were dissolved in DMSO-d_6_ and D_2_O, respectively and lyophilised three times from their respective solvent to exchange deuterium. ^13^C NMR spectroscopy was performed at 400 MHz and 40°C with approximately 15000 scans. ^31^P NMR spectroscopy was performed at 161.97 MHz and 40°C with 64 scans. Similarly, ^31^P NMR analysis of the milled sodium hexametaphosphate was performed. Before analysis, sodium hexametaphosphate was milled alone by planetary ball miller and then dissolved in D_2_O and lyophilised three times from D_2_O to exchange deuterium.

### Cell culture of murine macrophage RAW264.7 cell line

The murine macrophage cell line, RAW264.7, was cultured in DMEM supplemented with 100 U/mL penicillin, 100 µg/mL streptomycin, and 10% foetal bovine serum. Cells were grown in a 96-well plate at 37°C in a humidified 5% CO_2_ incubator [32]. RAW264.7 cells were harvested with a trypsin/EDTA solution (Gibco, USA). The cells were counted and plated into 96-well plates at 5×10^5^ cells/mL. Viability was routinely >97%. Cells were incubated overnight prior to beginning experiments.

### Cell proliferation of RAW264.7 cells

The effect of β-D-glucan phosphate on the viability of RAW264.7 cells was determined using the [3-(4,5-dimethylthiazol-2-yl)-2,5-diphenyltetrazolium] bromide (MTT) assay [32]. After pre-incubating RAW264.7 cells (5×10^5^ cells/mL) for 12 h, β-D-glucan phosphate or native β-D-glucan (50, 100, or 500 µg/mL) or lipopolysaccharides (LPS, 0.01, 0.1, or 1.0 µg/mL) were added, and the mixture was incubated for an additional 48 h. Twenty microlitres of the MTT stock solution (5 mg/mL) was then added to each well to attain a total reaction volume of 200 µL. After incubation for 4 h, the plate was centrifuged at 2000 rpm for 5 min, and the supernatants were aspirated. The formazan crystals in each well were dissolved in 150 µL DMSO, and the absorbance at 570 nm was recorded on a Molecular Plus-384 ELISA reader (Molecular, USA). Three wells were used for each GP concentration, and three independent experiments were performed.

### Neutral red uptake by RAW264.7 Cells

The ability of GP to improve the phagocytosis of RAW264.7 cells was determined by neutral red uptake analysis. After the pre-incubation of RAW264.7 cells (5×10^4^ cells/mL) for 12 h, each β-D-glucan phosphate or native β-D-glucan (50, 100, or 500 µg/mL) or LPS (0.01, 0.1, or 1.0 µg/mL) was added, and the mixture was incubated for an additional 24 h. The supernatant was then removed from the plate. Two hundred microlitres of fresh medium containing 0.01% neutral red was added into each well, and the cells were cultured for an additional 1 h. After 1 h, the cell plate was washed three times with PBS, and 200 µL of cell lysis buffer (acetic acid/ethanol; 1∶1) was added into each well, which was then kept at 4°C overnight [33]. The absorbance at 540 nm was detected using a Molecular Plus-384 ELISA reader.

### Determination of cytokines (TNF-α and IL-6) production and NO secretion in RAW264.7 cells

The ability of GP to induce cytokines (TNF-α and IL-6) production and NO secretion in RAW264.7 cells was determined using a commercial ELISA kit and Griess assay, respectively. After pre-incubating RAW264.7 cells (5×10^5^ cells/mL) for 12 h, each β-D-glucan phosphate or native β-D-glucan (50, 100, or 500 µg/mL) or LPS (0.01, 0.1, or 1.0 µg/mL) was added, and the mixture was incubated for an additional 48 h. The concentration of TNF-α and IL-6 in culture supernatants was determined using an ELISA kit (eBioscience, USA), according to the manufacturer’s instructions. The NO content of the culture supernatants was measured by adding 50 µL of Griess reagent (1% sulphanilamide and 0.1% N-[1-naphthyl]-ethylenediamine dihydrochloride in 2.5% phosphoric acid) to 50-µL samples. The NO concentration was determined at 540 nm using NaNO_2_ as a standard [32].

### Statistical analysis

Results were expressed as the means ± SD. A one-way analysis of variance (ANOVA) was performed for data analysis using SPSS (Statistical Package for Social Sciences) (10.0) software. Significant differences were indicated at *p*<0.05, whereas highly significant differences were indicated at *p*<0.01.

## Results

### Preparation of GP by planetary ball milling

**Figure 1:**
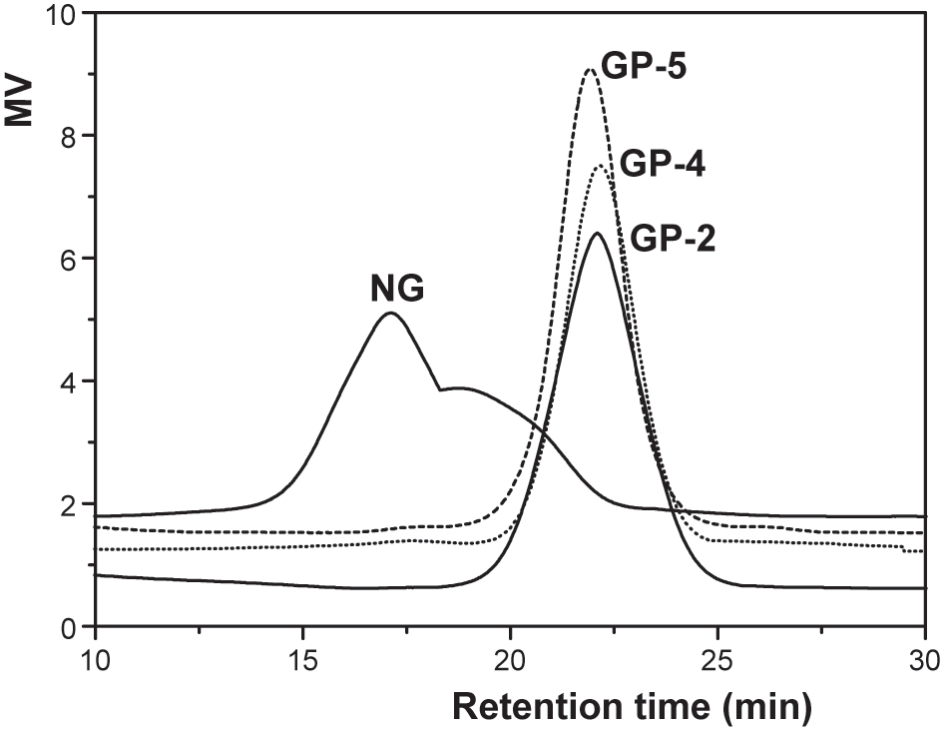
HPLC chromatogram of native β-D-glucan NG and three β-D-glucan phosphates, GP-2, GP-4, and GP-5 prepared by planetary ball milling.

The β-D-glucan particles isolated from *S. cerevisiae* were insoluble in water and weakly dissolved in DMSO [11]. After milling, the apparent PEAK molecular weight of all of the GPs prepared decreased greatly to 6.6–10.0 kDa (Table 1), much lower than that of the initial β-D-glucan material (430 kDa) (Fig. 1). Meanwhile, the water solubility and DS of each GP sample were also different, in accordance with the operating conditions (Table 1). Among the three operation parameters, the rotational speed of ball milling affected the water solubility of the GP products to the greatest extent, likely due to the different mechanical forces acting on the materials. The water solubility of GP-2 (78%) prepared at a rotational speed of 186.0 rad/s was much higher than that of GP-1 (54%) prepared at a speed of 139.5 rad/s (Table 1). Therefore, the ball-milling process was performed at a rotational speed of 186.0 rad/s in the following experiments.

**Table 1:** β-D-glucan phosphates prepared under different operation conditions.

Sample	Rotationalspeed (rad/s)	Millingtime (min)	Chargeratio^a^(g/g)	Solubilityin water (%)	DS (molP/mol Glc)	Apparent PEAKmolecularweight (Da)	DP	P* (KJ/g)
GP-1	139.50	12	1∶4	54±2.05	ND	10000	ND	3.03
GP-2	186.00	12	1∶4	78±2.11	1.24±0.013	8800	30.5	7.19
GP-3	186.00	16	1∶4	85±1.97	1.39±0.011	8500	28.0	9.58
GP-4	186.00	20	1∶4	84±3.42	2.09±0.175	6600	17.6	11.98
GP-5	186.00	20	1∶2	83±1.73	0.77±0.003	8100	33.7	11.98
GP-6	186.00	20	1∶6	84±0.98	1.95±0.002	9400	26.0	11.98

a weight ratio of β-D-glucan : sodium hexametaphosphate [(NaPO_3_)_6_].

ND not detected.

As the milling time increased from 12 min to 20 min, the water solubility of the GP products increased slightly, from 78% for GP-2 to 85% for GP-3 and 84% for GP-4, whereas the DS of the GP products increased significantly, from 1.24 for GP-2 to 2.09 for GP-4 (Table 1), indicating that the milling time has a significant impact on the efficiency of the phosphorylation reaction. Meanwhile, the DP of the GP products decreased from 30.5 for GP-2 to 17.6 for GP-4. In addition, the charge ratio of β-D-glucan to sodium hexametaphosphate also affects the phosphorylation of β-D-glucan. When β-D-glucan was co-milled with sodium hexametaphosphate at weight ratios of 1∶2, 1∶4, and 1∶6, respectively, at a rotational speed of 186.0 rad/s for 20 min, the DS of the GP products reached 0.77, 2.09, and 1.95, respectively, whereas the DP of the GP products decreased to 33.7, 17.6, and 26.0, respectively (Table 1), indicating that 1∶4 is a suitable charge ratio for producing GP with a much higher DS. Meanwhile, the polysaccharide yield after this phosphorylation process was about 70%, with the yield during the milling process 100% and that during the subsequent dialysing process 70%. Therefore, when operating under suitable conditions, soluble GP with a lower molecular weight and high DS could be successfully synthesised by the proposed mechanochemical method.

### Energy transferred during the milling process

To estimate the amount of energy transferred to synthesised materials via milling and to reveal the mechanochemical mechanism underlying this process, several methods have been employed to model the dynamics of planetary ball milling, such as the particle element method, the use of analytical/numerical models, and the discrete element method [21]. Among the various models, the one described by Magini and coworkers has been well accepted [22]. The following equation was deduced from this model to calculate the power P* transferred to a unit mass of milled powder [34]. The values predicted by this equation show good agreement with experimental observations made with respect to milling ball motion and energy transfer.
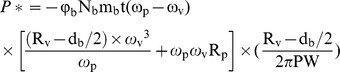



According to the above equation, the energy transferred to a milled powder depends on the number of balls (*N_b_*), the angular velocity of the vial (*ω_v_*) and plate (*ω_p_*), the geometrical parameters of the planetary ball-milling machine (*R_v_*, *R_p_*, and *d_b_*), and the milling time (*t*). In our experiment, the weight of the powder *PW* = 15 g; the number of milling balls *N_b_* = 1935; the weight of one ball *m_b_* = 7.80×10^−5 ^kg; the milling time *t* = 720, 960, or 1200 s; the angular velocity of the plate *ω_p_* = 117.00 or 87.75 rad/s; the angular velocity of the vial *ω_v_* = 186.00 or 139.50 rad/s; the distance from the centre of the plate to the centre of the vial *R_p_* = 5.20×10^−2 ^m; the radius of the vial *R_v_* = 3.50×10^−2 ^m; the diameter of the milling balls *d_b_* = 3.00×10^−3 ^m; and the empirical factor related to the degree of filling of the vial *φ_b_* = 0.87. Among these parameters, the angular velocity and milling time are the two most important variables, with the former affecting the transferred energy and the resulting mechanochemical reaction more greatly than the latter.

When milling for 12 min, the transferred power P* increased from a value of 3.03 KJ/g for a rotational speed (i.e., *ω_v_*) of 139.50 rad/s to 7.19 KJ/g for a rotational speed of 186.00 rad/s (Table 1). Accordingly, the water solubility of the corresponding GP product increased significantly. When the rotational speed was set to 186.00 rad/s, the transferred power P* increased linearly from 7.19 KJ/g for a milling time of 12 min to 11.98 KJ/g for a milling time of 20 min. Although the water solubility of three corresponding GP products was similar (approximately 80%), the DS of these GPs increased from 1.24 to 2.09, whereas the DP of these GPs decreased from 30.5 to 17.6, indicating that through varying the energy to some extent, GP products with different DS and DP could be produced and the energy provided by the planetary ball-milling machine was sufficient to induce the phosphorylation reaction between β-D-glucan and sodium hexametaphosphate as well as depolymerisation of β-D-glucan. However, although the energy transferred to GP-4, GP-5, and GP-6 was same, the DS of these three GPs varied significantly, indicating that besides the energy, other factors such as charge ratio would also influence the efficiency of phosphorylation reaction.

### Chemical characterisation of GP

**Figure 2:**
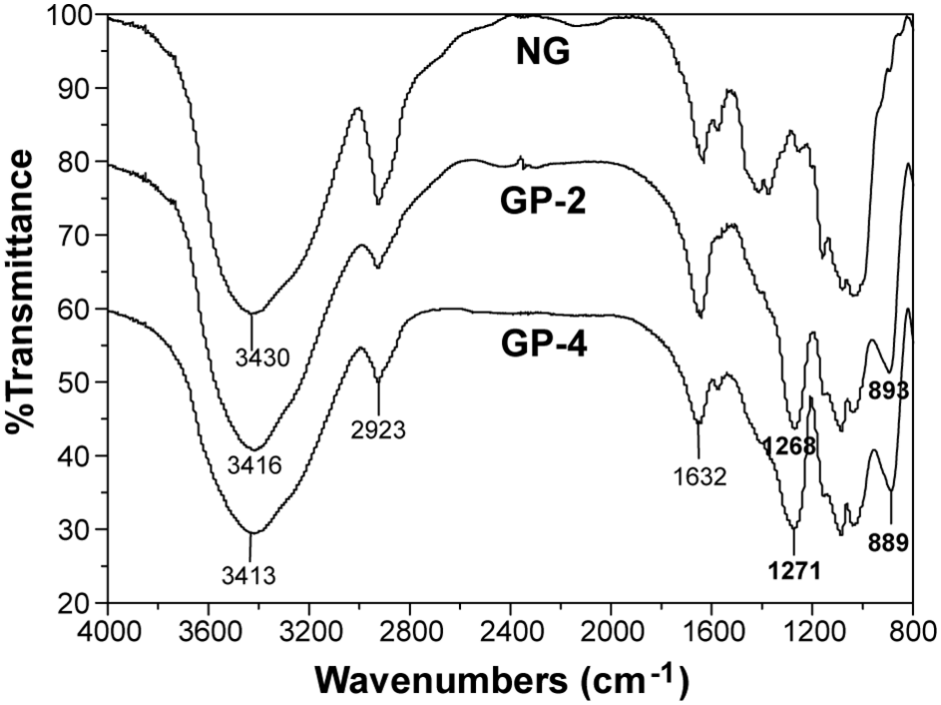
FTIR spectra of insoluble β-D-glucan particles NG and soluble β-D-glucan phosphate GP-2 and GP-4 prepared by planetary ball milling.

The structural changes in GP were monitored by FTIR as well as ^13^C and ^31^P NMR spectroscopy. The FTIR spectra of the initial insoluble β-D-glucan particles NG and the soluble derivatives GP-2 and GP-4 are compared in Fig. 2. The spectrum of untreated β-D-glucan is identical to that of β-D-glucan reported elsewhere [35], with the strong peak at 3430 cm^−1^ attributed to O–H stretching, the peak at 2923 cm^−1^ attributed to C–H stretching, and a peak at 1632 cm^−1^ attributed to the bending of bound water molecules. Two new peaks appeared in the spectra of GP-2 and GP-4: a strong peak at 1268 or 1271 cm^−1^ corresponding to the P = O bond and a slightly weaker peak at 893 or 889 cm^−1^ corresponding to the P–O bond, indicating that mechanochemical phosphorylation between β-D-glucan and sodium hexametaphosphate occurred during planetary ball milling.

**Figure 3:**
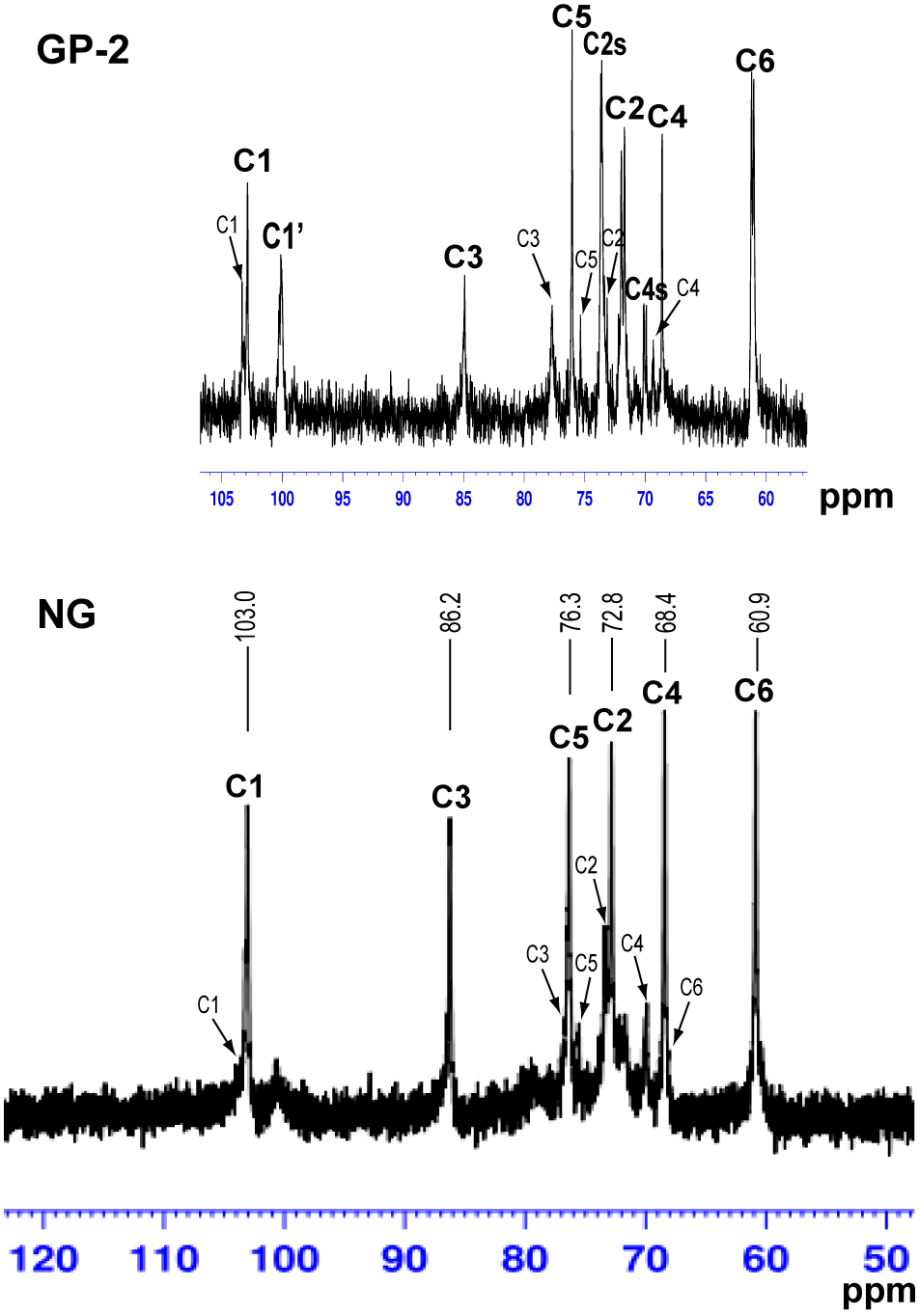
^13^C NMR spectra of insoluble β-D-glucan particles NG and β-D-glucan phosphate GP-2 prepared by planetary ball milling.

The ^13^C NMR spectrum of the initial insoluble β-D-glucan particles NG in DMSO-d_6_ (Fig. 3) is identical to those of *S. cerevisiae* β-D-glucans reported elsewhere [5], [36]–[38]. The initial insoluble β-D-glucan contains β-(1→3)- and β-(1→6)-linked D-glucosyl units, the former component being the predominant one. The ^13^C NMR spectrum of NG contains the signals corresponding to the carbon atoms of both types of units (Fig. 3). The main peaks at 103.0, 86.2, 76.3, 72.8, 68.4, and 60.9 ppm are assigned to the C1, C3, C5, C2, C4, and C6 of β-(1→3)-linked D-glucosyl units [5], [36], [37]. While the small peaks at 103.3, 76.5, 75.8, 73.2, 69.8, and 68.1 ppm can be attributed to the C1, C3, C5, C2, C4, and C6 of β-(1→6)-linked D-glucosyl units [36], [38]. The ^13^C NMR spectrum of the soluble β-D-glucan phosphate GP-2 (DS 1.24) in D_2_O showed the presence of new peaks (Fig. 3). The main peaks at 102.9, 84.9, 76.1, 71.7/72.0, 68.6, and 61.2/61.0 ppm are assigned to the original C1, C3, C5, C2, C4, and C6 of β-(1→3)-linked D-glucosyl units. While the small peaks at 103.3, 77.7, 75.4, 73.2, and 69.3 ppm can be attributed to the original C1, C3, C5, C2, and C4 of β-(1→6)-linked D-glucosyl units. A strong new peak appeared at 73.6/73.7 ppm in GP-2 is assigned to the substituted C2 (marked as C2s), whereas a small new peak at 70.1/69.9 ppm is designated as the signal of weakly substituted C4 (marked as C4s), because the chemical shift of substituted carbon will move into downfield and partial substitution will widen the peaks of substituted carbon [39]. This is similar to those observed in sulfated derivatives of (1→3)-β-D-glucan with DS of 1.14–1.74 [40] and sulfated derivative of α-(1→3)-D-glucan with DS of 1.4 [41], although the downfield shift of a carbon atom linked by a sulfate group there is 7–10 ppm and here by a phosphate group is about 2 ppm. Moreover, a new peak at 100.1 ppm can be assigned to C1 (marked as C1′) because C2 was substituted and thus influenced the chemical shift of the adjacent C1 [39]. Therefore, phosphate groups have been chemically bound to β-D-glucan.

**Figure 4:**
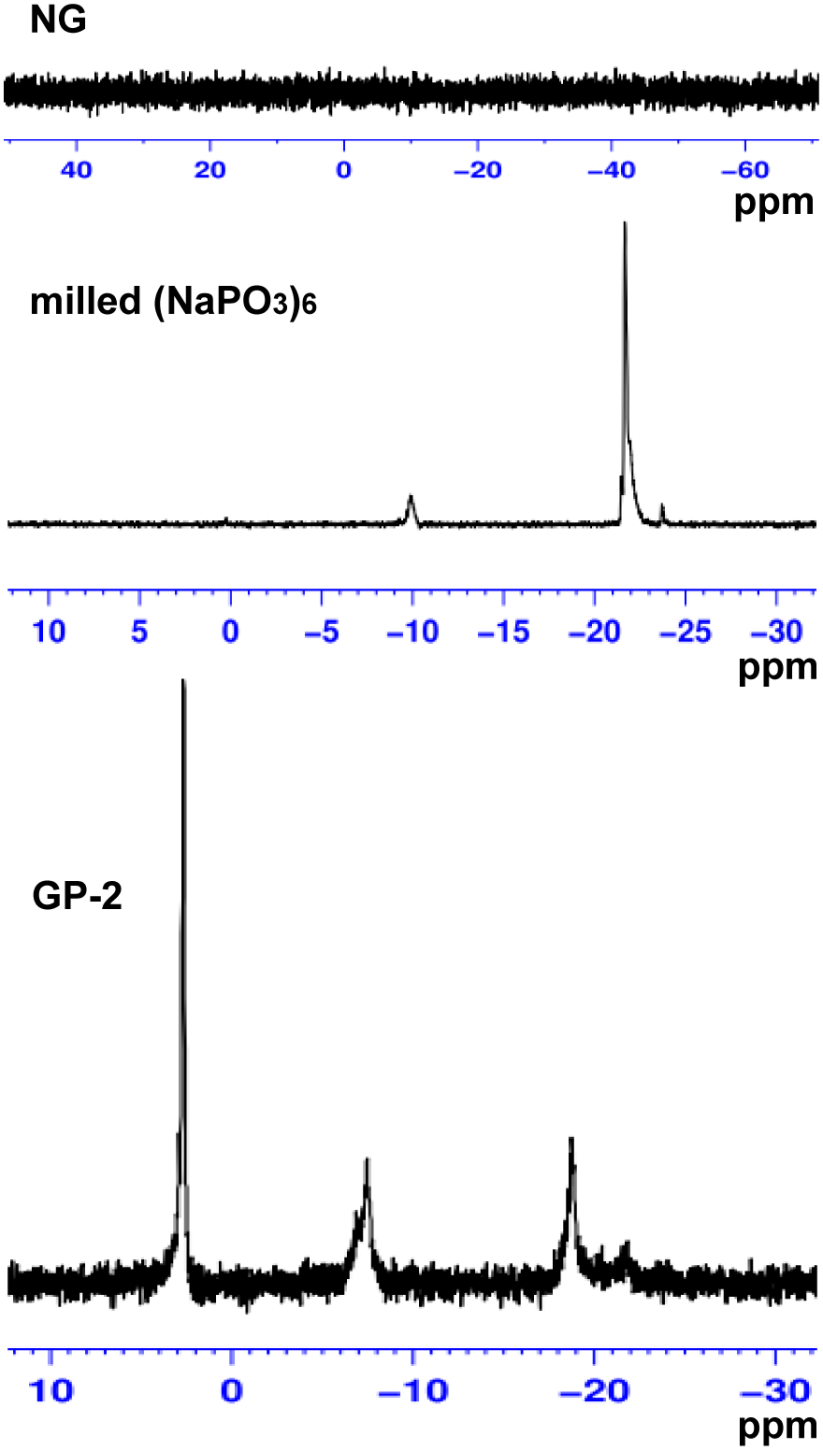
^31^P NMR spectra of insoluble β-D-glucan particles NG, β-D-glucan phosphate GP-2 prepared by planetary ball milling, and sodium hexametaphosphate (NaPO_3_)_6_ milled alone.

Further, ^31^P NMR spectral analysis of the initial insoluble β-D-glucan particles NG in DMSO-d_6_ and the soluble derivative GP-2 in D_2_O was undertaken to confirm the presence of the phosphate group in GP product. No signal was observed in NG, whereas a strong phosphorus signal was observed at 2.69 ppm in GP-2 (Fig. 4), similar with previously reported β-D-glucan phosphate [5], [42] as well as phosphated (1→3)-α-D-glucan [43], confirming that phosphate groups were bound to the molecular chain. In addition, two small signals were observed at –7.44 and –18.75 ppm, suggesting that other types of phosphate group were present in the phosphated β-D-glucan derivative, perhaps being minor portions of polysaccharide diphosphates and oligophosphates, as reported in starch phosphates [30]. This was further demonstrated by a weak signal at –9.87 ppm and a strong signal at –21.66 ppm observed in ^31^P NMR spectrum of sodium hexametaphosphate milled alone [milled (NaPO_3_)_6_] (Fig. 4). Therefore, in view of the ^13^C and ^31^P NMR results, phosphate groups have been chemically bound to β-D-glucan.

### Immunostimulating properties of GP on macrophage RAW264.7 cells

Macrophages play an important role in innate and adaptive immunity and are considered the main target cells of β-D-glucans. Macrophages act as the first line of immune defence by phagocytosing environmental particles and pathogens, recruiting and activating inflammatory cells, and producing a variety of pro-inflammatory cytokines and mediators, such as TNF-α, ILs, and NO, to regulate their own activity and the activity of other immune cells [44]. To examine whether the mechanochemically produced GP was able to stimulate the functional activation of macrophages, murine macrophage cells of the line RAW264.7 were incubated with 50–500 µg/mL of GP product or native β-D-glucan, and cell proliferation, neutral red uptake, cytokines (TNF-α and IL-6) production, and NO secretion were measured and compared to the amount produced by the untreated control group. Three GP products with different DS and DP, i.e., GP-2 with a DS of 1.24 and DP of 30.5, GP-4 with a DS of 2.09 and DP of 17.6, and GP-5 with a DS of 0.77 and DP of 33.7, were measured.

### Effect of GP on the proliferation of macrophage RAW264.7

**Figure 5:**
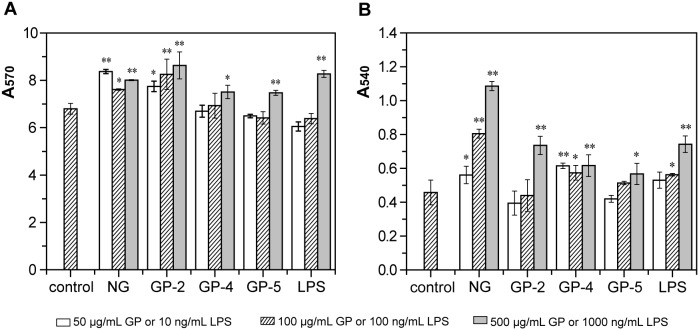
Effects of native β-D-glucan and three β-D-glucan phosphates, GP-2, GP-4, and GP-5, on cell proliferation (A) and neutral red uptake (B) of RAW264.7 cells. RAW264.7 cells were treated with NG or GP (50, 100, and 500 µg/mL) or LPS (10, 100, and 1000 ng/mL) in different concentrations as described in the Materials and Methods. After incubation, the viability of RAW264.7 cells was measured by an MTT assay, and the A_570_ value was recorded, whereas the amount of neutral red uptake was detected by the A_540_ value. The data represent the means ± SD. *p<0.05, **p<0.01 compared with control. Each point represents the average of three independent experiments.

First, the cytotoxic effect or proliferation effect of three GP products on RAW264.7 cells was examined. The MTT assay indicated that GP-2, GP-4, and GP-5 as well as NG and LPS enhanced the proliferation of RAW264.7 cells in a concentration-dependent manner, among which GP-2 exerted the most significant proliferation enhancement effect (114%, 121%, and 127% at concentrations of 50, 100, and 500 µg/mL), which was nearly same with the native β-D-glucan (Fig. 5A). GP-4 and GP-5 only enhanced the proliferation of RAW264.7 cells significantly at 500 µg/mL (*p*<0.05 and *p*<0.01 respectively).

### Effect of GP on the phagocytosis capacity of RAW264.7 cells

The amount of neutral red uptake by macrophages was measured to analyse the phagocytic ability of RAW264.7 cells incubated with GP products and native β-D-glucan. The phagocytosis of RAW264.7 cells increased significantly after being stimulated by a higher concentration (500 µg/mL) of GP-2, GP-4, and GP-5, with GP-2 inducing the greatest extent of phagocytosis (161%), which was equal to that stimulated by 1 µg/mL of LPS (Fig. 5B). However, a lower concentration of GP-4 also significantly stimulated the phagocytosis of RAW264.7 cells (50 µg/mL, *p*<0.01; 100 µg/mL, *p*<0.05), whereas a lower concentration of GP-2 and GP-5 did not. But the native β-D-glucan could stimulate the phagocytosis of RAW264.7 cells at each concentration, in a concentration-dependent manner.

### Effect of GP on the TNF-α and IL-6 production and NO secretion of RAW264.7 cells

**Figure 6:**
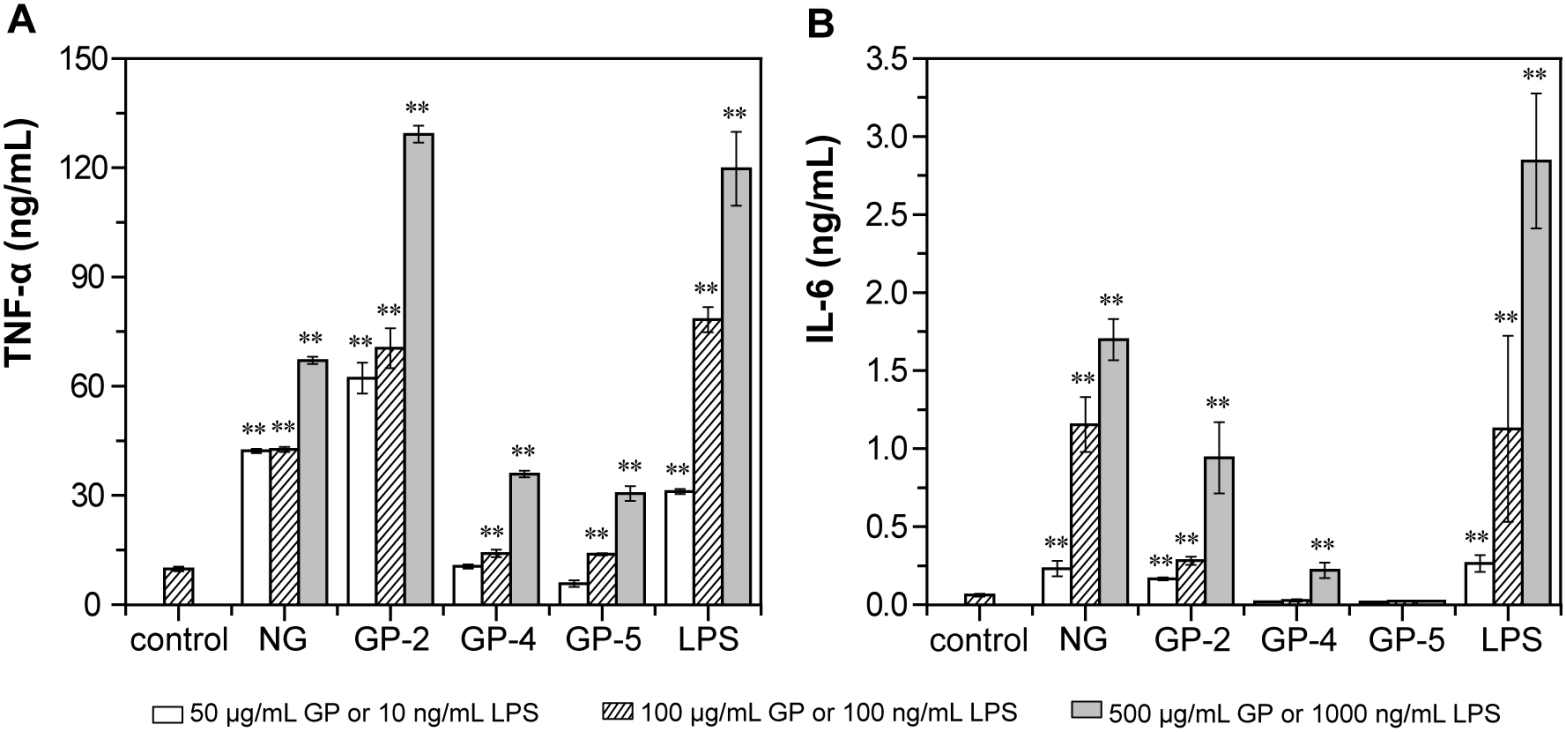
Effects of native β-D-glucan and three β-D-glucan phosphates, GP-2, GP-4, and GP-5, on TNF-α (A) and IL-6 (B) production by RAW264.7 cells. RAW264.7 cells were treated as Fig. 5. After incubation, the TNF-α and IL-6 concentrations in the supernatant were detected using commercial kits. The data represent the means ± SD. *p<0.05, **p<0.01 compared with control. Each point represents the average of three independent experiments.

Finally, the secretion of two major cytokines, TNF-α and IL-6, as well as a major inflammatory mediator, NO, by RAW264.7 cells incubated with GP was measured. TNF-α is an important host defence molecule that affects tumor cells. All three GP products and NG, as well as the positive control LPS, significantly stimulated TNF-α production in a concentration-dependent manner (Fig. 6A). The TNF-α production induced by GP-2 was much higher than that induced by GP-4 and GP-5, and also obviously higher that induced by NG. After being stimulated with 50, 100, and 500 µg/mL of GP-2 for 48 h, TNF-α production in the culture supernatant increased to 6.3-, 7.2-, and 13.1-fold that of the control, respectively. However, GP-4 and GP-5 stimulated TNF-α production significantly only at a concentration of 100–500 µg/mL (*p*<0.01), inducing a level of production 1.4–3.6- and 1.4–3.1-fold that of the control.

IL-6 is a major cytokine involved in B cell differentiation, T cell activation, induction of acute phase proteins, and reduction of the G0-residence time of hematopoietic cells. NG, GP-2, and GP-4, as well as LPS, induced IL-6 secretion in RAW264.7 cells in a concentration-dependent manner, with GP-2 stimulating a higher level of secretion than GP-4 and a lower level of secretion than NG, whereas GP-5 suppressed IL-6 secretion (Fig. 6B). After being stimulated with 50, 100, and 500 µg/mL of GP-2, IL-6 production in the culture supernatant increased significantly to 2.6-, 4.5-, and 14.9-fold that of the control, respectively. GP-4 only induced the secretion of IL-6 significantly at a concentration of 500 µg/mL (*p*<0.05).

**Figure 7:**
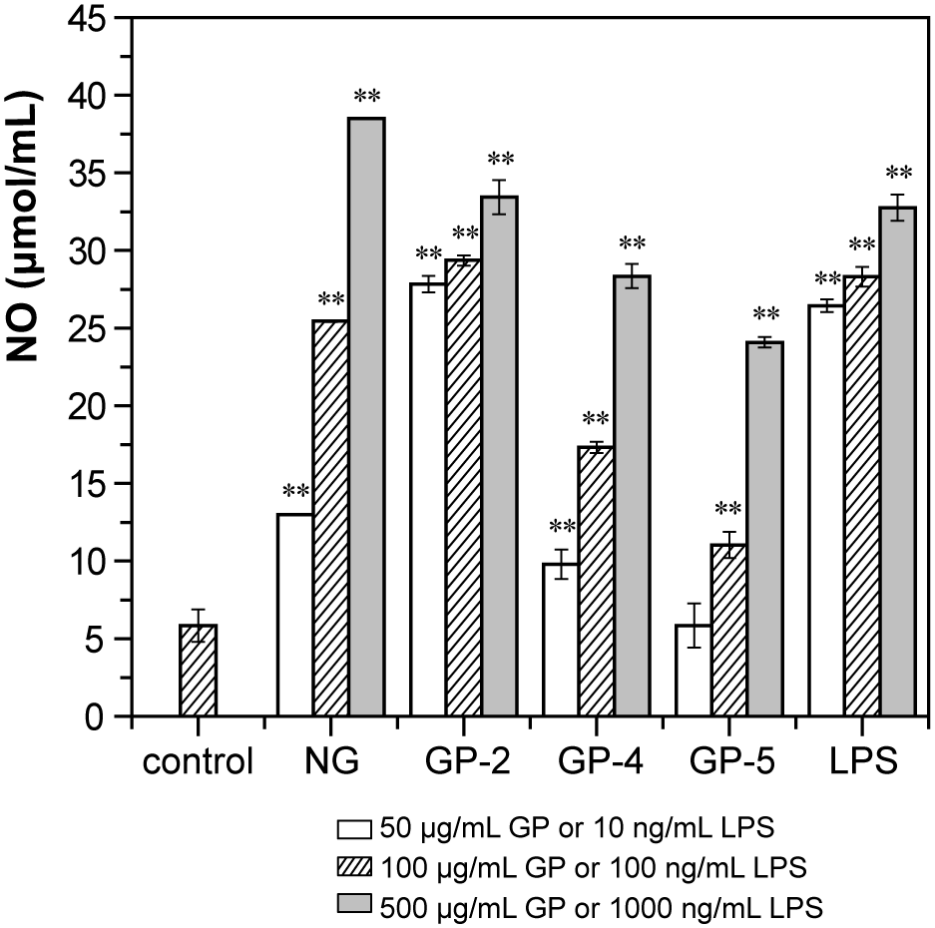
Effects of native β-D-glucan and three β-D-glucan phosphates, GP-2, GP-4, and GP-5, on NO secretion by RAW264.7 cells. RAW264.7 cells were treated as Fig. 5. After incubation, the NO concentrations in the supernatant were detected by the A_540_ value after reacting with Griess reagent. The data represent the means ± SD. *p<0.05, **p<0.01 compared with control. Each point represents the average of three independent experiments.

NO is a major mediator of macrophages that acts as a destroyer to bacteria and tumor cells. A Griess assay indicated that all three GP productss and native β-D-glucan, as well as LPS, induced secretion of NO significantly in a concentration-dependent manner, with GP-2 triggering the highest level of NO secretion and GP-5 the lowest among the three GPs, whereas NG similar with GP-2 (Fig. 7). After being stimulated with 50, 100, and 500 µg/mL of GP-2, the concentration of NO in the culture supernatant increased to 4.8-, 50-, and 5.7-fold that of the control, respectively. After being stimulated with 50, 100, and 500 µg/mL of GP-4, the amount of NO secreted increased to 1.7-, 3.0-, and 4.9-fold that of the control, respectively. On the other hand, GP-5 only triggered NO secretion significantly at concentrations of 100 and 500 µg/mL (*p*<0.01). Therefore, among the three GP products, GP-2 with a DS of 1.24 and DP of 30.5 exhibited the strongest immunostimulating activity and was nearly similar with NG.

## Discussion

The application of an environmentally friendly, solvent-free, and convenient method, mechanochemical processing, to phosphorylate and solubilise β-D-glucan isolated from brewer’s yeast, *S. cerevisiae*, was investigated in this study. Water-soluble β-D-glucan derivatives, β-D-glucan phosphates, with a greatly reduced apparent PEAK molecular weight of 6.6–10.0 kDa and high DS of 0.77–2.09 as well as greatly decreased DP of 17.6–33.7 were successfully prepared by planetary ball milling (Table 1). The result indicated that the tight helical structure of β-D-glucan molecules could be broken and activated to react with sodium hexametaphosphate by mechanical force. Moreover, the phosphorylation efficiency of the planetary ball-milling process was very high because the reaction could be completed within 20 min, indicating that mechanical energy was effectively transformed into the internal energy of the milled materials. This transformed energy could improve the chemical reactivity of the hydroxyl groups of β-D-glucan molecule and increase the number of metastable active sites, with which the sodium hexametaphosphate could rapidly react. When the insoluble β-D-glucan was depolymerised and modified with the hydrophilic phosphate groups by this process, its water solubility improved significantly. Most importantly, compared to the traditional phosphorylation process of β-D-glucan, which is carried out in an organic solvent at elevated temperature [5], our process is convenient, clean, solvent-free, and easy to implement. The phosphorylation reaction could be performed by a one-step process. Therefore, it may be favourable for environmental and industrial application.

The presence of phosphate groups in the GP products was demonstrated by FTIR spectroscopy (Fig. 2) as well as ^13^C and ^31^P NMR spectroscopy (Fig. 3 and 4). The obtained GP products were biologically active and able to upregulate the functional events mediated by activated macrophage cells of the RAW264.7 line, such as cell proliferation, phagocytosis, cytokines (TNF-α and IL-6) production, and NO secretion (Fig. 5–7), especially TNF-α production and NO secretion, with GP-2 (DS = 1.24, DP = 30.5) exerting the highest immunostimulating activity, which was similar with the native β-D-glucan.

However, according to previous reports, the immunity-modulating activity of yeast β-D-glucans depends on the molecular weight, glycosidic linkages, and chemical groups attached to the main chain. Phosphated β-D-glucan with a DS of 0.14 prepared by traditional chemical modification in an organic solvent has been reported to exert immunity-modulating activity [13], [14]. β-D-glucan sulfate with a DS of 1.14 and molecular weight of 46 kDa has been reported to exhibit the highest anti-tumor activity among several sulfated derivatives [16]. Carboxymethylated β-D-glucan derivative with DS of 0.75 showed the highest mitogenic activity, while derivatives with DS higher than 1.0 were inactive [17]. These researches demonstrate that the appropriate DS and chemical group influences the activity of β-D-glucan. In this study, a comparison of GP products with the similar apparent PEAK molecular weight (6.6–10.0 kDa) and various DS and DP demonstrated that GP-2 with a DS of 1.24 and DP of 30.5 exerted higher biological activity than GP-4 with a higher DS (2.09) and lower DP (17.6) and GP-5 with a lower DS (0.77) and higher DP (33.7) (Fig. 5–7), suggesting that the appropriate DS of phosphate groups and the appropriate DP of β-D-glucan are the two main determinants of immune cell stimulation. Meanwhile, the biological properties of GP-2 were similar with those of the initial β-D-glucan particles, among which the TNF-α secretion of RAW264.7 cells stimulated by GP-2 were even a little higher than that by native β-D-glucan. The reason for the different immunostimulating activities of these GP products will be researched further.

In conclusion, mechanochemical processing can be used as a simple, efficient, and clean method to produce biologically active GP, which may be attractive for industrial application.
